# A High Kinetic Energy Ion Mobility Spectrometer for Operation at Higher Pressures of up to 60 mbar

**DOI:** 10.1021/jasms.2c00365

**Published:** 2023-03-31

**Authors:** Florian Schlottmann, Christoph Schaefer, Ansgar T. Kirk, Alexander Bohnhorst, Stefan Zimmermann

**Affiliations:** Institute of Electrical Engineering and Measurement Technology, Leibniz University Hannover, Appelstr. 9a, 30167 Hannover, Germany

## Abstract

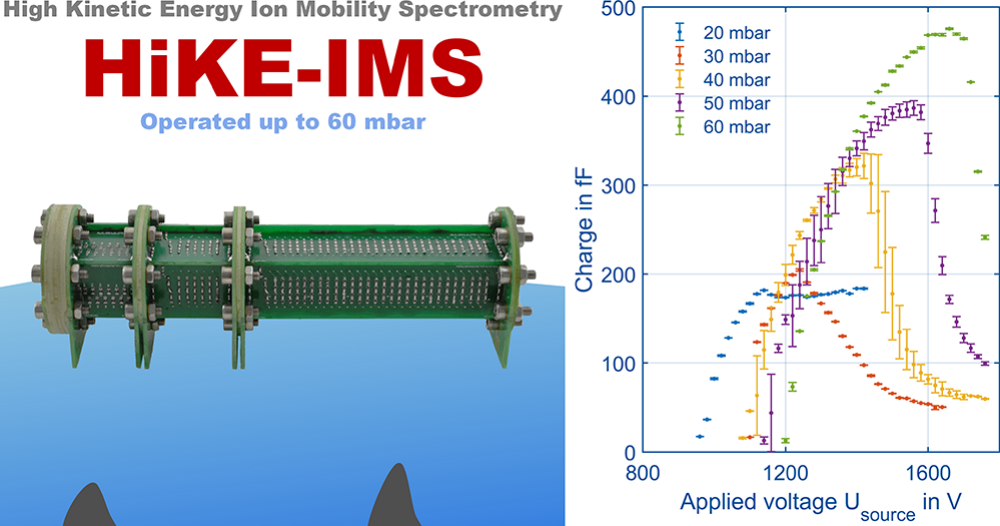

High Kinetic Energy Ion Mobility Spectrometers (HiKE-IMS) are usually operated at absolute pressures around 20 mbar in order to reach high reduced electric field strengths of up to 120 Td for influencing reaction kinetics in the reaction region. Such operating points significantly increase the linear range and limit chemical cross sensitivities. Furthermore, HiKE-IMS enables ionization of compounds normally not detectable in ambient pressure IMS, such as benzene, due to additional reaction pathways and fewer clustering reactions. However, operation at higher pressures promises increased sensitivity and smaller instrument size. In this work, we therefore study the theoretical requirements to prevent dielectric breakdown while maintaining high reduced electric field strengths at higher pressures. Furthermore, we experimentally investigate influences of the pressure, discharge currents and applied voltages on the corona ionization source. Based on these results, we present a HiKE-IMS that operates at a pressure of 60 mbar and reduced electric field strengths of up to 105 Td. The corona experiments show shark fin shaped curves for the total charge at the detector with a distinct optimum operating point in the glow discharge region at a corona discharge current of 5 μA. Here, the available charge is maximized while the generation of less-reactive ion species like NO_*x*_^+^ is minimized. With these settings, the reactant ion population, H_3_O^+^ and O_2_^+^, for ionizing and detecting nonpolar substances like *n*-hexane is still available even at 60 mbar, achieving a limit of detection of just 5 ppb_V_ for *n*-hexane.

## Introduction

For fast online and onsite measurements of trace gases in ambient air, ion mobility spectrometry is an often used technique.^1,2^ Ion mobility spectrometry provides limits of detection in the low ppb_V_ (parts-per-billion by volume) and even ppt_V_ (parts-per-trillion by volume) range^1^ within measurement times of 1 s. Therefore, ion mobility spectrometers (IMS) are mainly used in safety and security applications, e.g., for the detection of chemical warfare agents,^3−5^ toxic industrial chemicals,^6,7^ drugs,^8−10^ or explosives.^11−13^ In an IMS, the formed ions are separated by their individual drift motion in a drift region being the acceleration in an applied electric field and repeated deceleration through collisions with neutral gas molecules. In most cases clean, dry air is used as neutral gas at an operating pressure of about 1000 mbar. Typical ionization sources are for example weak radioactive sources, e.g., ^3^H, ^63^Ni, or ^241^Am.^1^ Corona discharge ionization source as commonly used for atmospheric pressure chemical ionization (APCI) can also be used in IMS instead of radioactive materials.^14^ The large number of collisions at pressures of about 1000 mbar makes these ionization methods very efficient, and polar substances or such with high proton affinity can be detected with high sensitivity. Unfortunately, nonpolar substances with low proton affinity are difficult or impossible to detect due to the higher proton affinities of the conjugated base of the prevailing protonated water clusters. Other drawbacks of IMS operated at around 1000 mbar are the low linear range and strong matrix effects.^15−19^ The generated ion population is in thermodynamic equilibrium and thus does not represent the actual sample gas composition.

The aforementioned issues can be reduced or eliminated when using special devices like a High Kinetic Energy Ion Mobility Spectrometer (HiKE-IMS), which is built similarly to an ambient pressure ion mobility spectrometer but operated at an absolute pressure around 20 mbar.^20^ Reduced pressures enable using high reduced electric field strengths ε = *E*/*N* of up to 120 Td (Townsend; 1 Td = 10^–21^ Vm^2^) in the reaction and separation region, with the electric field strength *E* divided by the neutral molecule density *N*. Therefore, ε is a measure for the energy an ion acquires while accelerating in between two collisions. In addition, high reduced electric field strengths lead to higher drift velocities of the ions and therefore to reduced reaction times in the reaction region in the order of 100 μs to 1 ms. These short reaction times result in kinetic control, not reaching a thermodynamic equilibrium and hereby a heavy decrease of the cross sensitivities.^21^ Additionally, high reduced electric field strengths enable dissociation of ion-neutral clusters allowing to detect even substances with a low proton affinity and nonpolar substances by bare H_3_O^+^ and O_2_^+^. Furthermore, HiKE-IMS allows the observation of additional orthogonal parameters related to an increased ion temperature such as fragmentation, declustering, and field-dependent ion mobility, which help to separate compounds in the separation region that have similar ion mobility under low field conditions. Ion mobility spectrometers at 1000 mbar reach only reduced electric field strengths of 2 Td, which are considered as low field conditions.^1^ However, compared to IMS, sensitivity of HiKE-IMS is reduced for compounds with high proton affinity and high dipole momentum due to reduced collision rates at low HiKE-IMS pressure.

The above-mentioned benefits of HiKE-IMS with respect to substance ionization are also known from other devices like proton transfer reaction mass spectrometers (PTR-MS) or selected ion flow drift tube mass spectrometers (SIFDT-MS) to control the chemical ionization processes at decreased pressures. Recent publications of Allers et al. have focused on the reactant and product ion formation in HiKE-IMS^22−25^ which showed the same mechanisms as described among others by Španěl,^26−28^ Good,^29^ Kebarle,^30−32^ and Zhao.^33^ Despite the similarity in ionization mechanisms, the HiKE-IMS ionizes, separates, and detects substances in a reaction and separation region operated and same pressure, e.g., at 20 mbar, while PTR-MS and SIFDT-MS ionize in a low vacuum at 2 mbar and detect substances in a high vacuum,^34−36^ requiring large and power intensive vacuum pumps that make operation in field applications challenging. Thus, HiKE-IMS provides promising miniaturization potential compared to SIFDT-MS or PTR-MS, as HiKE-IMS are evacuated by a single membrane pump,^20^ which is beneficial for future hand-held instrumentation and field application.

Since the pressure of the HiKE-IMS is not necessarily fixed at 20 mbar, other operating pressures have been used to understand, e.g., fundamentals of corona discharge ionization or product ion generation in HiKE-IMS covering a range from 7 mbar^37^ up to 40 mbar.^38^ Especially, ref (38) showed a significant improvement of sensitivity and thus limits of detection by changing the operating pressure from 20 to 40 mbar, while still reaching similar reduced electric field strengths. It was shown that sensitivity increases in a quadratic manner with operating pressure, indicating that even higher operating pressures would be desirable. Furthermore, if the operating pressure of HiKE-IMS would be higher, even smaller and lighter vacuum pumps could be used, which again benefit the development toward future hand-held instrumentation. All this leads to the question if HiKE-IMS operation is possible at even higher pressure with similar high reduced electric field strengths. However, as the reduced electric field strength should reach the same maximum value as reported in ref (38) while pressure is increased, the electric field has to be increased likewise to maintain the reduced electric field strength. In this case, it is mandatory to guarantee that increasing static electric field strengths do not lead to dielectric breakdown. This is more challenging compared to dynamic fields, such as in ref (39), since a breakdown is less probable for high frequency sinusoidal voltages. Reaching high reduced static electric field strengths will be discussed in the next section of this paper. As also known from the literature, e.g., refs (14) or (40), increasing the operating pressure affects corona discharge ionization. This will be investigated experimentally with a newly designed, further miniaturized HiKE-IMS that is to be operated at a maximum pressure of now 60 mbar.

## Theoretical Considerations on Dielectric Breakdown in HiKE-IMS

The most important design considerations concern the electrode arrangement in the reaction and separation region in order to prevent dielectric breakdown at the desired high reduced electric field strength and at the intended high pressure. For this purpose, it may be easier to consider the electric field strengths *E* resulting from the reduced electric field strengths ε or, ultimately, the voltage *U* applied between two adjacent electrodes in the HiKE-IMS reaction and separation region. The relation between the three parameters is shown in eq 1 which also includes the neutral density *N* and the length *L* across which the voltage *U* is applied. Eq 1 can also be solved for the voltage required to reach a certain ε as shown in eq 2, which is necessary for, e.g., estimating the dielectric breakdown voltage between two adjacent electrodes. Furthermore, by replacing *N* with the pressure *p* divided by the temperature *T* and the Boltzmann constant *k*_B_, eq 2 is derived. The voltage *U* depends linearly on pressure *p*, length *L*, and reduced electric field strength ε, which will be important in the following.
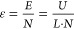
1
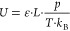
2

**Figure 1:**
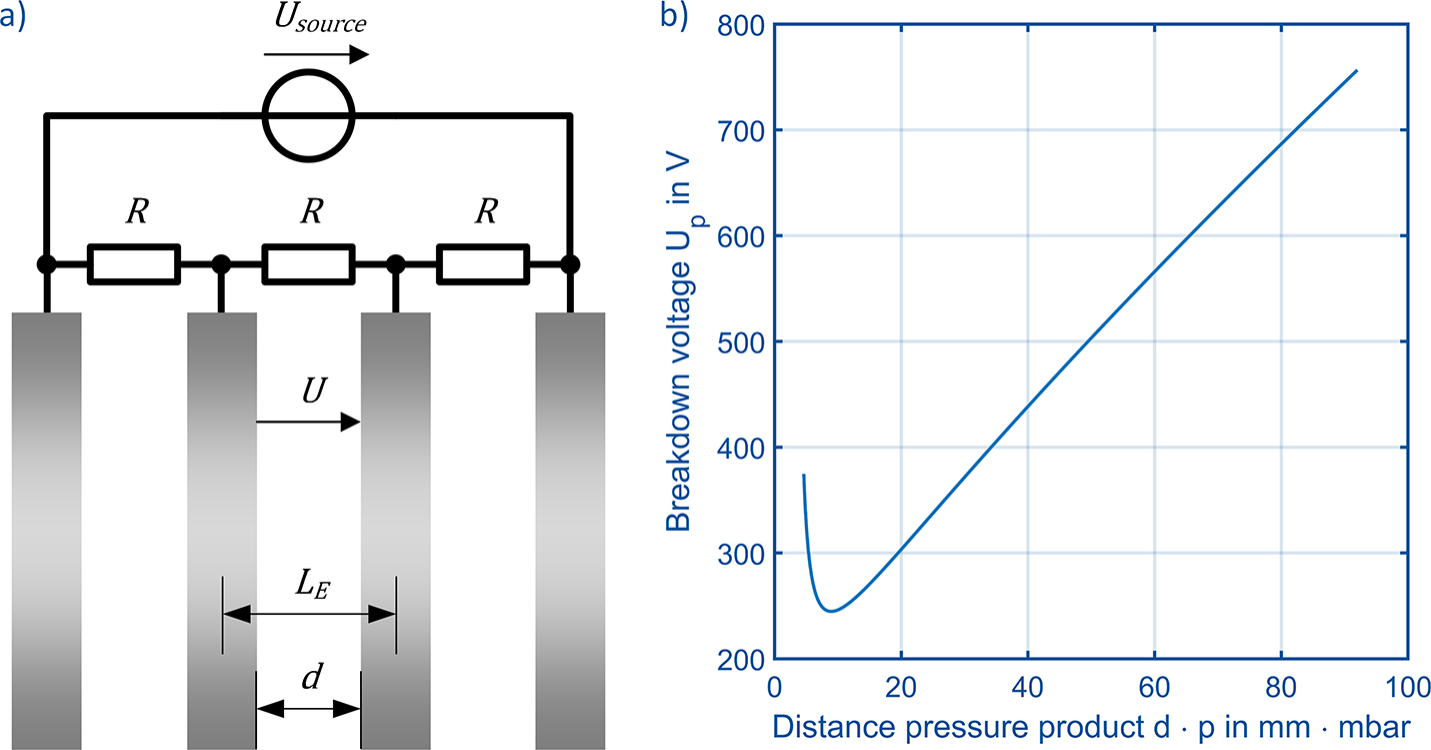
(a) Schematic of four drift electrodes of a HiKE-IMS with the different geometrical parameters, the voltage applied, and the resistors and voltage source connected to the electrodes. (b) Paschen’s curve taken from ref (41), with *A* = 1130 (mm bar)^−1^, *B* = 27.4 kV/(mm bar), and γ = 0.025.

In Figure 1a, the different geometric or size related parameters affecting possible dielectric breakdown are shown: the smallest distance *d* in between two electrodes, which is the most critical size for dielectric breakdown, and the center distance between two electrodes, the length *L*_*E*_, needed for calculating the voltage *U*. For preliminary estimation whether dielectric breakdown is possible, Paschen’s law and its analytical approximations can be used, see eq 3 and Figure 1b. It is important to note that eq 3 assumes two parallel electrodes forming a plate capacitor with a homogeneous electrical field between the two electrodes. In particular corner effects are not considered. Eq 3 gives information about the breakdown voltage *U*_*P*_ of an electrode arrangement as a function of pressure *p* and distance *d* between two adjacent electrodes. The other influencing parameters *A*, *B*, and γ depend on the gas between the electrodes and the electrode material.^41^ Typically, *U*_*P*_ is plotted over the distance pressure product *d*·*p*. When increasing pressure at an arbitrarily chosen distance pressure product of 20 to 40 mm·mbar at a constant distance *d* of 1 mm and thus pressures from 20 to 40 mbar, the breakdown voltage increases in a linear manner. Nevertheless, the breakdown voltage increases from 302 V at 20 mm·mbar to 440 V at 40 mm·mbar. Since we aim for the same reduced electric field strengths ε, the voltage *U* needs to double when the pressure doubles and the distance *d* remains constant, as shown in eqs 1 and 2. However, this conflicts with the breakdown voltage *U*_*P*_ which just increases from 302 V at 20 mm·mbar to 440 V at 40 mm·mbar. If applying the voltage *U* needed to reach the same reduced electric field strength ε, which is *U* = 604 V, *U* exceeds *U*_*P*_ and dielectric breakdown becomes possible. As a result of this theoretical consideration, electrode design in drift tubes, in this case for the reaction and separation region for high pressure HiKE-IMS, needs to be considered carefully to not exceed *U*_*P*_.
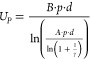
3

Not only the distance pressure product, but also other well-known factors like geometry of the electrodes and even space charges can have an impact on *U*_*P*_.^42^ Additionally, small distances in the μm regime between two electrodes can result in other curves for *U*_*P*_. More detailed discussions are available, e.g., in refs (43−46). Furthermore, creepage currents across the isolating surface between two electrodes can occur and afflict HiKE-IMS experiments. Therefore, we recommend to stay as far as possible below the minimum of the Paschen curve and to choose the distance *d* between the electrodes greater than or equal to 500 μm as used, e.g., in refs (47, 48).

The general condition used for designing HiKE-IMS or similar devices is stated in eq 4 and shows *U* has to be smaller than *U*_*P*_; here *L* has been replaced by *L*_*E*_ as adjacent electrodes are considered. If this condition is violated, starting from the first initial breakdown between two adjacent electrodes, an avalanche-like discharge can follow through the entire device. Each dielectric breakdown can cause irreversible damage to various elements of the periphery, for example, the transimpedance amplifier or tightly designed power supplies. This could be observed in the course of the past years and during the experiments conducted in HiKE-IMS.
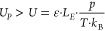
4When dividing eq 4 through eq 3, the breakdown condition in eq 5 is established. Most importantly, if the pressure *p* is increased at constant reduced electric field strength ε, length *L*_*E*_ and distance *d* can be tuned so that the right term stays below 1 and dielectric breakdown is avoided. However, the distance *d* has always to be smaller than the length *L*_*E*_, as otherwise no electrode material would remain.
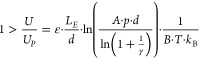
5

**Figure 2:**
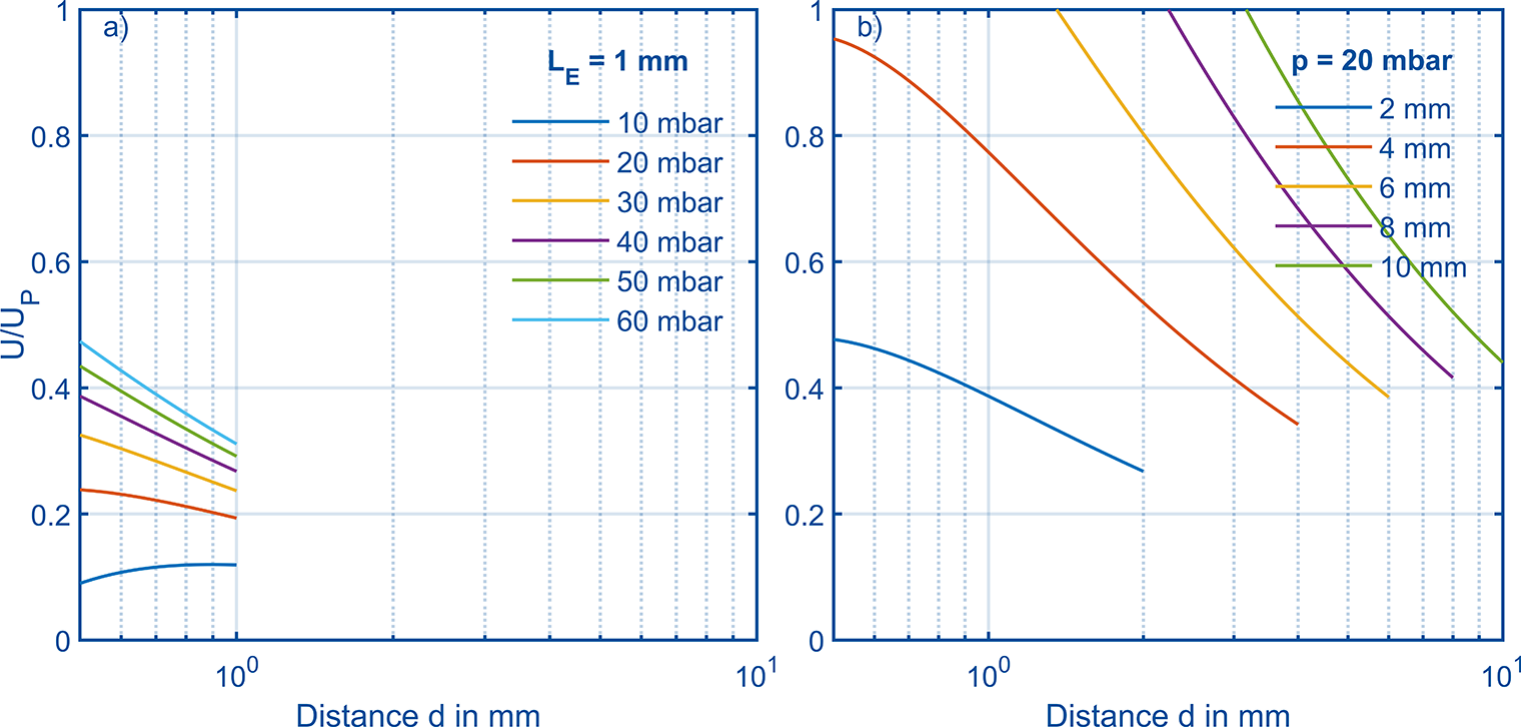
(a) *U*/*U*_*P*_ over possible electrode distance *d* at various pressures and constant length *L*_*E*_ = 1 mm in order to reach *E*/*N* = 120 Td and to avoid dielectric breakdown. All curves end at a distance *d* = 1 mm as distances *d* ≥ 1 mm are physically impossible. (b) Possible electrode distance *d* at various length *L*_*E*_ and constant pressure *p* = 20 mbar in order to reach *E*/*N* = 120 Td and to avoid dielectric breakdown. Again, all curves end when the distance *d* reaches the center–center length between the electrodes.

Eq 5 can be plotted over geometric parameters of interest such as the distance between two electrodes. Focusing on *d* and *p* at a constant temperature *T* = 293.15 K and length *L*_*E*_ = 1 mm aiming at a reduced electric field strength ε = 120 Td, a pressure variation over distance *d* is performed, which is shown in Figure 2a. In this case, dielectric breakdown would be avoided at all pressures, as all values are below 0.5. Figure 2b shows a variation of the length *L*_*E*_ between the electrodes at constant pressure *p* of 20 mbar, because at higher pressures and given *L*_*E*_ varying d result in the right term exceeding 1 and dielectric breakdown is most likely going to happen. Even at 20 mbar, at values for *L*_*E*_ greater than 4 mm, the critical value of 1 is exceeded for small distances *d*. For example following the yellow curve for length *L*_*E*_ = 6 mm, the critical value of 1 is exceeded for distances *d* smaller than 1.37 mm, but at distances *d* larger than 1.37 mm operation would theoretically be possible. Considering that, with a rather large length *L*_*E*_ the distance *d* required to operate at the HiKE-IMS at high reduced electric field strength and at high pressure has to be large as well. Small lengths like in the case of *L*_*E*_ = 2 mm allow various distances *d* to reach high reduced electric field strength at high pressure. In addition, choosing a large length *L*_*E*_ can also result in electric field inhomogeneity inside the IMS. Therefore, for high pressure HiKE-IMS an electrode design that has as many electrodes as possible resulting in a small length *L*_*E*_ and maximum distance *d* is most favored, giving additional benefits in electric field distribution.^49^ Additional information about how the electrode design affects IMS drift tube performance, e.g., resolving power or field distribution, is found in ref (49). In general, we propose the usage of printed circuit boards (PCB) as these allow the design and manufacturing of a large number of electrodes with a small length *L*_*E*_ and almost any desired distance *d*.

## Experimental Section

**Figure 3:**
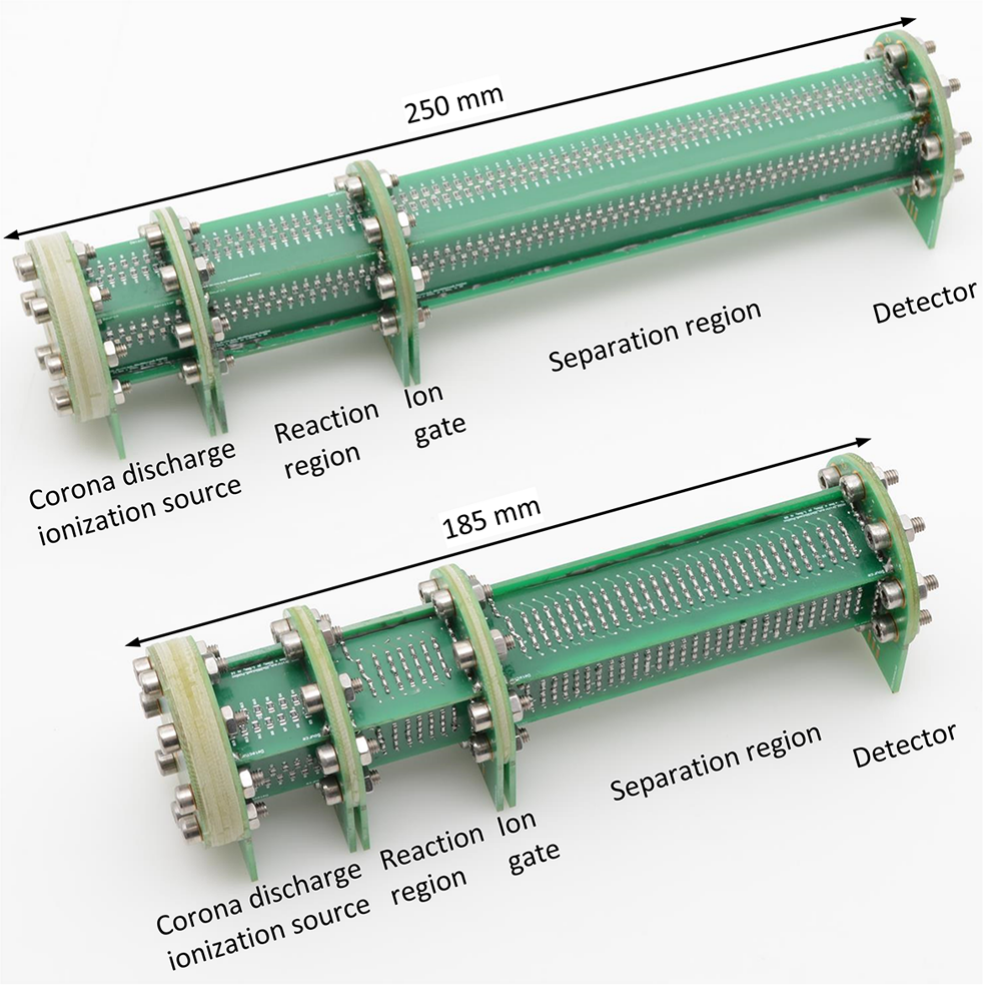
Two different PCB-based HiKE-IMS: HiKE-IMS identical to ref (38), optimized for operation at 40 mbar (top), and HiKE-IMS used in this work, optimized for operation at 60 mbar (bottom).

With respect to the theoretical considerations presented in this paper we verified the electrode design from Bohnhorst et al.^48^ (*d* = 0.5 mm and *L*_*E*_ = 1.5 mm), whether the electrode design is able to withstand high reduced electric field strengths of up to 120 Td at an operating pressure of 60 mbar. The maximum value of *U*/*U*_*P*_ is 0.711 at 60 mbar according to eq 4, which is below the condition for dielectric breakdown at the increased pressure. However, as pressure and therefore voltage applied to the electrodes increase, also the power dissipated in the resistor network has to be taken into account in order to prevent significant self-heating. In Figure 3 (top) a HiKE-IMS as used in ref (38) is shown. This HiKE-IMS utilizes the same electrode design as Bohnhorst et al.^48^ but with 10 MΩ resistors, where each resistor dissipates up to 2.25 mW at an electric field strength of 100 V/mm corresponding to a reduced electric field strength of 100 Td at the maximum operating pressure of 40 mbar. If the same HiKE-IMS with the same resistors is operated at identical reduced electric field strength but an increased pressure of 60 mbar, the electric field strength increases to 150 V/mm, more than doubling the power dissipation to 5.06 mW due to the quadratic dependence between power dissipation and voltage. Across a HiKE-IMS separation region with an arbitrarily chosen length of 100 mm with 67 resistors, and using 4 of these PCBs parallel to form a quadratic shaped HiKE-IMS, power dissipation would in total reach up to 1.36 W. In order to half self-heating and reach a similar level as in ref (38), the number of resistors between two rings is doubled (serial configuration) also utilizing smaller form factor resistors with identical resistance of 10 MΩ, as larger resistors with same accuracy (1%) and required electric strength are not available. Thus, power dissipation at the two resistors between two electrodes now reaches only 2.53 mW and 0.68 W across the whole separation region at the increased operating pressure. The HiKE-IMS used in this work considering the constraints presented here to operate the HiKE-IMS at a maximum pressure of 60 mbar while reaching the desired reduced electric field strengths of more than 100 Td is shown in Figure 3 (bottom). The length of the HiKE-IMS built in this work is scaled down in order to use same voltage sources as in refs (38) and (50), since the resolving power should remain the same, which depends on the total separation region voltage.^50^ Thus, the HiKE-IMS to be operated at 60 mbar has a shorter reaction and separation region compared to ref (38). However, the corona discharge ionization sources design was not changed. Both HiKE-IMS are built from PCBs, as shown in Figure 3, and the total length including corona discharge ionization source and the detector region is reduced from 250 mm to 185 mm.

**Figure 4:**
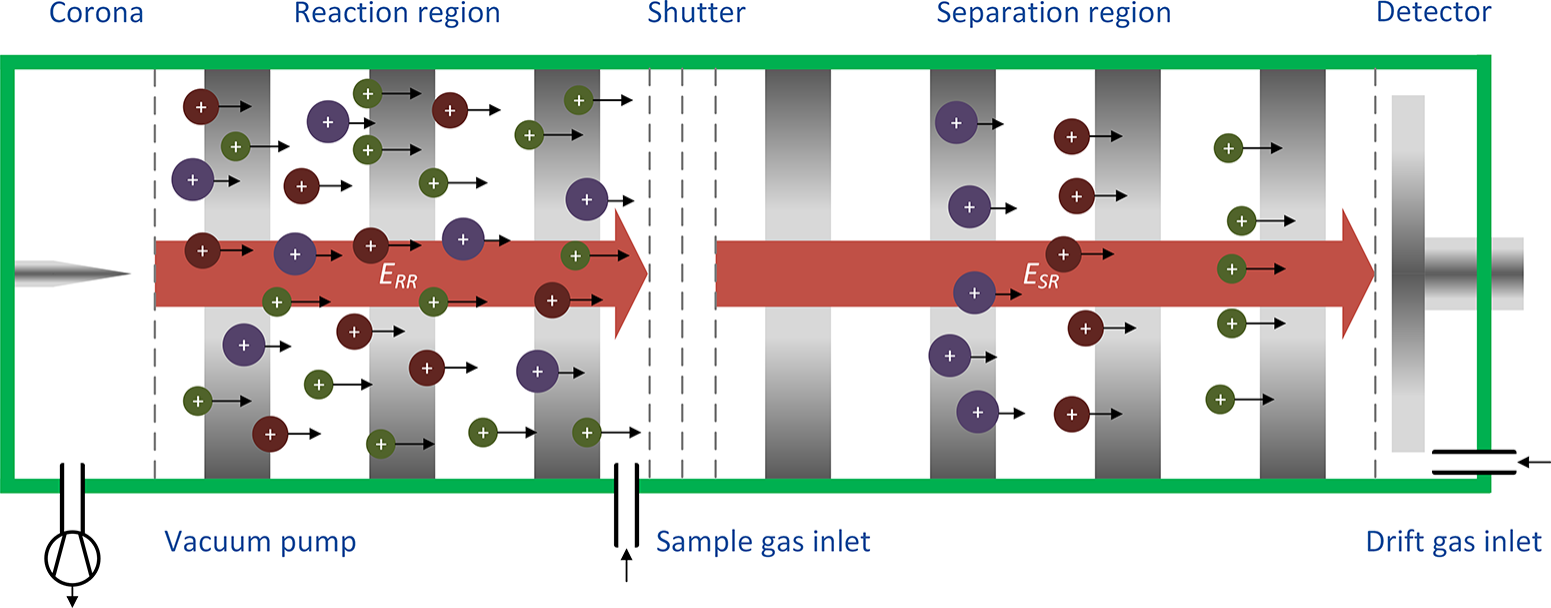
Schematic of a HiKE-IMS.

The schematic underlying both HiKE-IMS shown in Figure 3 is presented in Figure 4. A corona discharge ionization source consisting of a corona needle (Corona Needle APCI, Agilent Technologies, Australia) and an etched grid electrode generates primary ions. These primary ions react with the clean, dry air to form stable reactant ions which ionize substances in the reaction region. A tristate ion shutter as presented in ref (50) injects narrow ion packets into the separation region where the ions are separated by their ion mobility. The electric field strengths and thus the reduced electric field strength in the reaction region and the separation region can be adjusted individually. The detector is a simple Faraday plate. Drift and sample gas are directly fed into the HiKE-IMS from ambient pressure via flow restricting capillaries with 250 μm inner diameter and fixed lengths (1.4 m) to provide gas flow rates of 10 mL_s_/min (milliliter standard per minute, mass flow at reference conditions 20 °C and 1013.25 mbar) for both sample and drift gas. The drift gas purges the separation region and the reaction region and mixes within the reaction region with the sample gas. Purified, dry air (1.4 ppm_V_ water) is used for both the drift and the sample gas. Water concentrations are measured by a dew point sensor (Easidew Online, Michell Instruments, Germany). Pressure within the HiKE-IMS is monitored with a capacitive pressure gauge (SPOT CDS530D, Inficon, Switzerland). The HiKE-IMS is evacuated via an adjustable membrane pump (N84.4AN.29DC-B, KNF, Germany) which is driven by custom-built control electronics. Adjusting the pumping rate of the membrane pump from 100% down to 10% at the given flow rates leads to a relative pressure increase of 35 mbar. To cover a pressure range from 20 to 60 mbar another optional flow restriction between the HiKE-IMS and the membrane pump is used. In this work, the reduced electric field strength is kept constant at 70 Td in the reaction region and 100 Td in the separation region.

**Figure 5:**
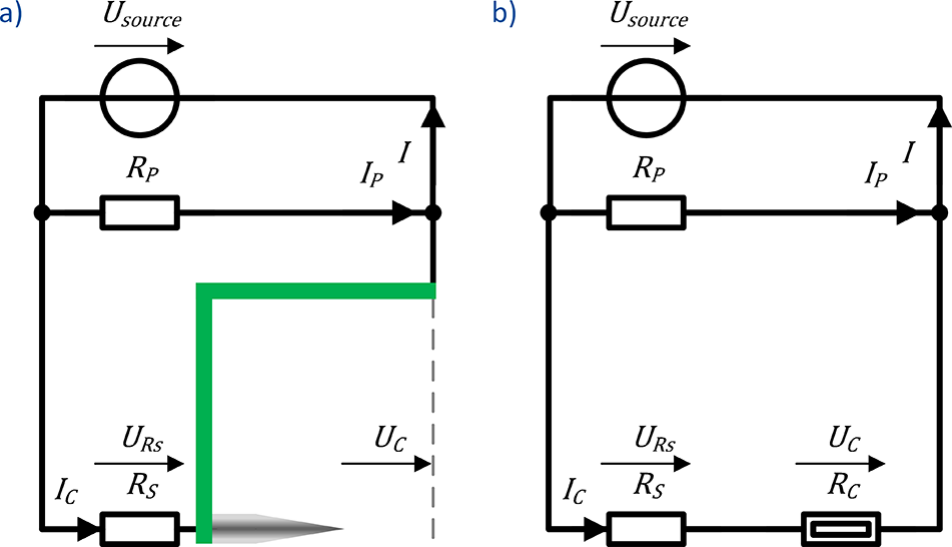
(a) Electric circuit diagram of the corona discharge ionization source with the resistor *R*_*S*_ in series with and *R*_*P*_ in parallel to the corona discharge. (b) Simplified circuit with the corona discharge ionization source as a nonlinear resistor.

The corona discharge ionization source is driven by a voltage source, making the characterization of the nonlinear behavior known from corona discharges^51^ more challenging. Therefore, the network of resistors shown in Figure 5a is used, with the parallel resistor *R*_*P*_ as a constant load to stabilize the voltage source. The series resistor *R*_*S*_ limits the maximum corona discharge ionization current *I*_*C*_. In Figure 5b, the schematically drawn needle-grid arrangement of the corona discharge ionization source is replaced by a nonlinear resistor *R*_*C*_ describing the corona discharge. When measuring the voltage of the voltage source *U*_source_ and its current *I*, both the corona discharge ionization current *I*_*C*_ and the corona discharge ionization voltage *U*_*C*_ are calculated according to eqs 6 and 7. Also, note that the charge at the Faraday plate is obtained from the numeric integral over the measured, 6400-times signal averaged ion mobility spectra. In this paper, however, we restrict ourselves exclusively to the positive polarity of corona discharge ionization, since the negative polarity implies additional challenges, such as an increased electron density in the HiKE-IMS reaction region increasing the probability for electrical breakdown and Trichel-pulses.^51^ An investigation of the negative polarity in HiKE-IMS at elevated pressure including the negative reactant and product ions will be part of a future publication.
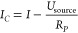
6
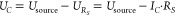
7

The electronics to drive the HiKE-IMS, such as the ion gate controller and the isolated voltage supply for the corona discharge ionization source, are reported in refs (38, 50). The ion current at the Faraday plate is amplified by a transimpedance amplifier with a bandwidth of 248 kHz and a gain of 45 MΩ, which is also designed and built at our institute.^52^ A digitizer (ADQ14DC-2A-USB, Teledyne SP Devices, Sweden) acquires the amplified ion mobility spectra. Additionally, a commercially available 20 kV voltage source (HCP 14-20000, FuG Elektronik, Germany) provides the separation region voltage. The HiKE-IMS is mounted in a custom-built, heated housing and kept at a constant temperature of 39.7 °C. All operational parameters are summarized in Table 1. For comparison, Table 1 also includes the geometric and operational parameters of previous HiKE-IMS publications from Langejuergen et al.,^21^ Kirk et al.,^50^ and Schlottmann et al.^38^ At this stage of development, the setup is a laboratory grade demonstrator for testing the components and exploring effects present at increased pressures. Furthermore, the experiments require the handling of very high DC voltages, in some cases more than 20 kV, which pose an acute danger. Therefore, trained and qualified personnel have set up, commissioned, and run all the experiments.

**Table 1:** Operational Parameters of the HiKE-IMS Used in This Work Compared to Previous Publications from Langejuergen et al.,^21^ Kirk et al.,^50^ and Schlottmann et al.^38^

parameter	value (this work)	value (from ref ^21^)	value (from ref ^50^)	value (from ref ^38^)
Reaction region length	34.8 mm	108 mm	77 mm	50 mm
Separation region length	101.5 mm	145 mm	306 mm	150 mm
Overall length (including corona discharge ionization source and connectors)	185 mm	310 mm	440 mm	250 mm
Inner dimension of the separation and reaction region	20 mm × 20 mm, rectangular shape	21 mm, circular shape	21 mm, circular shape	20 mm × 20 mm, rectangular shape
Voltage applied to the corona discharge ionization source	800–1800 V	1000 V	1200 V	1200–1550 V
Reaction region voltage	1.14–3.39 kV	1.5–6.05 kV	up to 5 kV	up to 5 kV
Reaction region reduced field	70 Td	30–120 Td	15–125 Td	85 Td
Separation region voltage	4.83–14.33 kV	0.65–7.4 kV	up to 20 kV	up to 20 kV
Separation region reduced field	100 Td	9–120 Td	15–125 Td	105 Td
Injection time	1 μs	6 μs	1–3 μs	3 μs
Resolving power	95	60 (100 Td)	135 (100 Td)	69
Number of averages	6400	1000	3600	1024
Averaging time	2.4 s	2 s	1.8 s (125 Td) to 18 s (15 Td)	0.512 s
Length of measured spectrum	381 μs	340 μs (120 Td) to 1400 μs	500 μs (125 Td) to 5000 μs (15 Td)	500 μs
Drift gas flow	10 mL/min	5.35 mL/min	10 mL/min	20 mL/min
Sample gas flow	10 mL/min	7 mL/min	10 mL/min	10 mL/min
Dew point of drift gas and sample gas	–74 °C (1.4 ppm_V_ water vapor concentration)	<1 ppm_V_ water	–90 °C (90 ppb_V_ water vapor concentration)	–74 °C (1.4 ppm_V_ water vapor concentration)
Pressure	20–60 mbar	19–20 mbar	20 mbar	20–40 mbar
Operating temperature	39.7 °C	35–35.5 °C	25 °C	25 °C
Resistor *R*_*S*_	22 MΩ	no information	no information	100 MΩ
Resistor *R*_*P*_	12.2 MΩ	no information	no information	15.2 MΩ

## Results and Discussion

**Figure 6:**
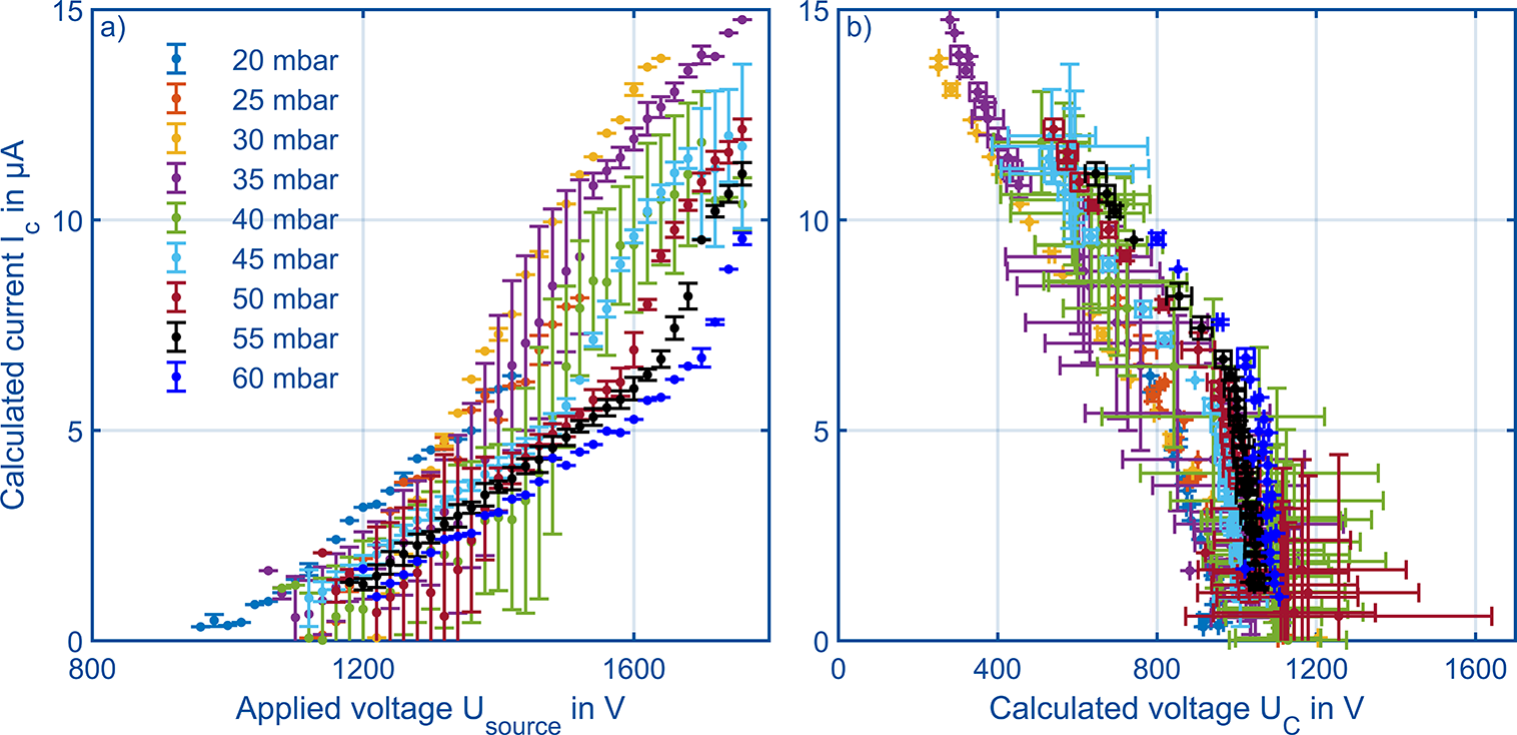
(a) Calculated current of the corona discharge based on Figure 5 over the voltage applied to the corona ionization sources at varying pressures from 20 to 60 mbar in air. (b) Calculated current of the corona discharge over the calculated voltage between the corona needle and the grid electrode. Table 1 summarizes all other operational parameters.

At first, in similar experiments to the well-known literature of corona discharge ionization sources, for example,^51^ the relation of the corona discharge ionization voltage *U*_*C*_ and the resulting current *I*_*C*_ has been investigated for the HiKE-IMS presented in this work. As shown in the legend of Figure 6a we varied the pressure in 5 mbar steps and the applied voltage *U*_source_ in steps of 20 V. Each step was held for at least 10 s while three data sets were stored including all relevant information and parameters like voltages, currents, and ion mobility spectra of the reactant ions. The resulting data points for each pair of applied voltage and pressure have been averaged and the error bars show the standard deviation of the calculated corona current. When considering the measurements in Figure 6a, a steady increase in discharge ion current for increasing applied voltage in all recorded data points is obvious, which is in agreement with the literature^51^ showing a constant conductivity between the corona needle and counter electrode, and thus, a constant charge density and mobility. In the literature, a stable glow region for corona discharges in positive polarity is described for currents between roughly 1 and 10 μA at atmospheric pressure.^51^ Exceeding the glow region results in formation of streamer discharges or even arcing, what is in practice a dielectric breakdown that can severely damage electronics. In agreement with that, our experiments show that corona currents *I*_*C*_ above 12 μA are not recommendable as the corona discharge is operated outside the stable glow region. For example, dielectric breakdown was observed at a pressure of 30 mbar exceeding the last stable point (applied voltage of 1640 V giving a calculated current of 13.7 μA). It has to be noted that the currents measured and calculated are the average currents, thus temporarily higher currents can occur. Figure 6b shows the actual corona voltage calculated from eq 6 and 7. Due to calculation of the actual corona voltage, Figure 6b shows a broader standard deviation of the data points in the direction of the abscissa. At corona currents above 5 μA, the calculated voltage *U*_*C*_ decreases, which is a strong indicator for unstable operation of the corona ionization source due to increasing space charge effects. This decline of the calculated voltage *U*_*C*_ agrees with the typical curves for corona ionization sources presented in ref (51). In the following figures the applied voltage will be used, as this is the parameter that is technically varied during measurements.

**Figure 7:**
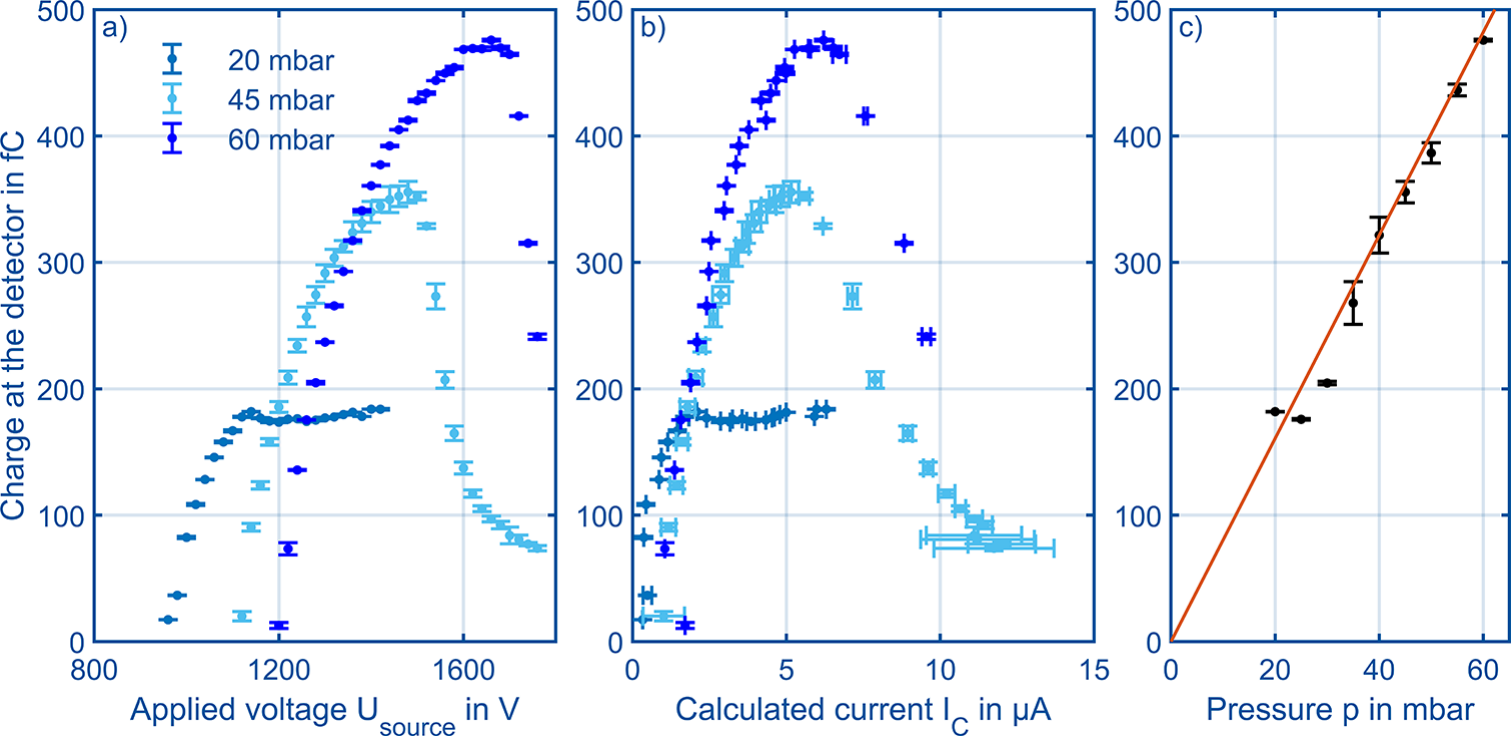
(a) Charge at the detector of the HiKE-IMS over the voltage applied to the corona discharge ionization source at varying pressure. All other parameters given in Table 1. (b) Charge at the detector of the HiKE-IMS over the current of the corona discharge ionization source at varying pressure. (c) Maximum charge at the detector of the HiKE-IMS over pressure validating the model from Kirk et al.^37^ even at higher pressures. Table 1 summarizes all other operational parameters.

Next, the available number of reactant ions is investigated, as the reactant ion density is crucial for the product ion generation rate and thus sensitivity.^38^ Here, the total number of reactant ions is studied via the total charge at the detector. Therefore, in Figure 7a, the total charge is plotted over the voltage applied to the corona discharge ionization source for exemplary pressures of 20, 45, and 60 mbar. All curves initially show an almost linear increase of the total charge with the applied voltage, thus increasing the corona discharge ionization source current *I*_*C*_ as already visualized in Figure 6a. At lower pressures of 20 and 25 mbar, the linear increase is followed by a plateau, which is a space charge driven effect that will be discussed in the next paragraph. For higher pressures between 30 mbar and 60 mbar, the curve resembles a shark-fin with a pronounced maximum. When operating at 30 mbar or higher and applying high voltage to the corona discharge source, a decline of the total charge at the detector is recorded. This can be explained by fast transients of the corona discharge in combination with signal averaging of the ion mobility spectra. Small streamer discharges occur at the corona discharge ionization source increasing the averaged corona discharge ionization source current *I*_*C*_. These streamer discharges are followed by a harsh decrease in actual corona voltage, as the voltage drop at the series resistor *R*_*S*_ increases and eventually interrupts the corona discharge. After the corona discharge stops, the corona ionization source current *I*_*C*_ is negligibly small, the corona discharge ionization voltage *U*_*C*_ increases, and the corona discharge starts again. This results in a periodic course leading to reduced total charges recorded at the detector due to signal averaging. An even higher applied voltage finally leads to dielectric breakdown as described above. Figure 7b shows the charge at the detector over the calculated corona discharge ionization sources current *I*_*C*_. Here, the maxima of the shark-fin shaped peaks are around 5 μA, showing that this maximum is almost independent from the pressure, as the main driving force behind corona discharge processes are the inhomogeneous electric fields and thus the reduced electric field strength close to the corona needle.^41,42^ Thus, operating the corona discharge ionization source is favored in this point.

However, also known from the literature,^37,38^eq 8 describes the influence of pressure in HiKE-IMS on the measured ion current at the detector. Here, length *L*_car_ is the characteristic length of the reaction region.^37^ Operating at constant reduced electric field strength ε inside a HiKE-IMS with fixed length *L*_car_ generating the same reactant ion species with identical reduced ion mobility *K*_0_, only the number of neutral gas particles *N* increases linearly with increasing pressure. Eq 8 is independent from the corona discharge ionization source current itself, as with ion current also charge density increases, leading to stronger Coulomb repulsion in radial direction and more ion discharge at the ring electrodes.^37^ Likely, this is causing the plateau of the measured charge at the detector at varying corona discharge currents. Plotting the maximum charge of each curve from Figure 7a and b over the pressure gives Figure 7c confirming this relation, here, for even higher pressures in a similarly constructed but shorter HiKE-IMS. Adding up to that, from Figure 7c it becomes clear that operation close to the maximum possible charge at the detector is desired; otherwise, as shown in ref (38), the number of generated product ions is influenced by absolute number of available reactant ions.
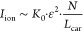
8

As different reactant ion species can ionize substances via different reaction pathways like proton transfer reaction (H_3_O^+^) or direct ionization (O_2_^+^ and NO_*x*_^+^), it is also important to know how the reactant ion population changes when changing operating parameters. Allers et al. coupled a self-built time-of-flight mass spectrometer to a HiKE-IMS and showed how reactant ion species formation depends on the reduced electric field strengths.^24^ By comparing peak positions in the ion mobility spectra, ion species are identified with respect to Allers et al.,^22−24,53^ who used a HiKE-IMS-MS. The four dominant reactant ion peaks in the new HiKE-IMS at elevated pressures are as expected NO^+^, H_3_O^+^, NO_2_^+^, and O_2_^+^. Table 2 lists the different reactant ions and their ion mobilities in air from the Supporting Information of ref (53) and compares these values to the values calculated across all pressures in this work. The agreement is very high, except for minor deviations for O_2_^+^, which can be explained by the different setups probably having different moisture levels, which may lead to cluster reactions. Another possible effect changing the O_2_^+^ ion mobility could be the oxygen concentration in the drift gas leading to resonant charge transfer as shown in refs (24, 53), which depends on air treatment and tubing and can thus slightly differ from setup to setup.

**Table 2:** Comparison of Ion Mobilities *K*_0_ Calculated in This Work with Ion Mobilities *K*_0_ from the Supporting Information of ref (53) at 100 Td in the Separation Region and at Water Concentration of 70 ppm_V_

reactant ion species	*K*_0_ in cm^2^/(V s) taken from ref (53)	*K*_0_ in cm^2^/(V s) in this work
NO^+^	3.031	3.02 ± 0.01
H_3_O^+^	2.801	2.80 ± 0.01
NO_2_^+^	2.620	2.62 ± 0.01
O_2_^+^	2.426	2.46 ± 0.01

**Figure 8:**
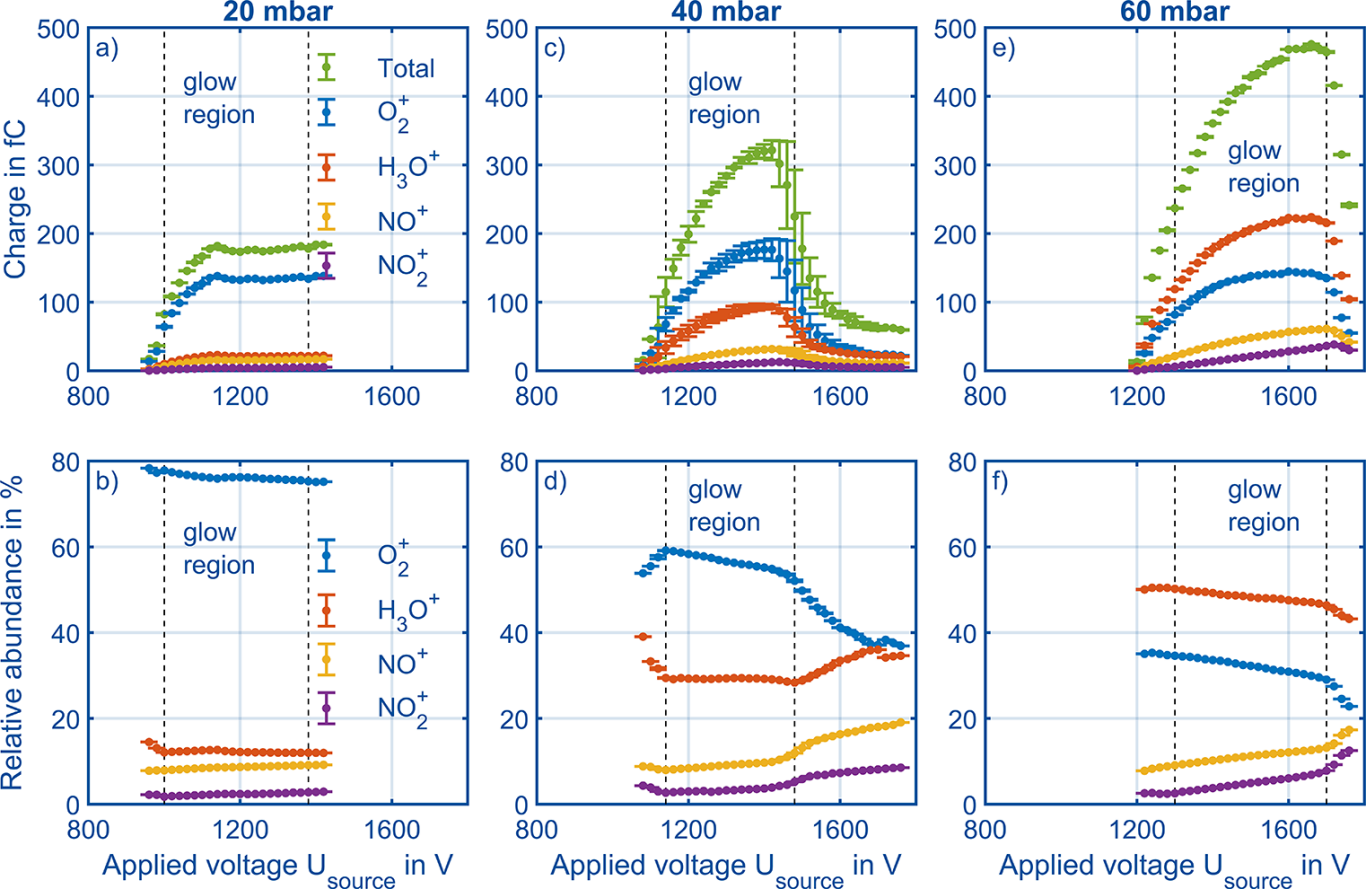
Total charge and charge of the four different reactant ion species at the detector at different pressures starting from 20 mbar (a and b), 40 mbar (c and d), and 60 mbar (e and f). Table 1 summarizes all other operational parameters.

At a pressure of 20 mbar, the absolute charge of each individual reactant ion species follows the curve of the total charge (green), see Figure 8a. The most abundant reactant ion at 20 mbar is O_2_^+^ (blue). Figure 8b shows the relative abundances with O_2_^+^ making nearly 80% of the total ion current. A more detailed consideration of the pressure dependence of the various reactant ion species is given in the next paragraph. The other three reactant ions have a share below 15% with a slight increase of NO_*x*_^+^ ions at increasing corona voltage. When increasing the pressure to 40 mbar at same reduced electric field strengths, Figure 8c and d result. For the absolute abundances, the behavior is quite similar to 20 mbar, with O_2_^+^ the most abundant ion, followed by H_3_O^+^. Again, the absolute abundance of each reactant ion species follows the total charge at the detector. However, the relative abundancies show a different course: H_3_O^+^ is decreasing from 40% to 30% relative abundance between 960 and 1140 V, stays at a plateau until 1500 V, and is then increasing again, showing the three distinct regions of a corona discharge: (1) first ignitions, (2) glow region, (3) beginning of streamer discharges. As the first region is unfavorable due to poor ion yield and the third due to instability and possible dielectric breakdown, the second region is where the corona discharge has to be operated. Furthermore, the increases of the relative NO_*x*_^+^ abundances indicate when the third region is reached.^54,55^ In the second region, or glow region, the abundance for NO_*x*_^+^ almost stays below 10%. Thus, a high relative amount of NO_*x*_^+^ in combination with a lower reactant ion current can indicate the region in which the corona discharge ionization source is operated. In Figure 8e and f, the pressure is increased further to 60 mbar. Here, H_3_O^+^ is the most abundant reactant ion, in absolute numbers in Figure 8e and in relative numbers in Figure 8f, which will be discussed later. Anyhow, there is a linear increase of NO_2_^+^ until the pronounced maximum is reached; all other reactant ions follow the total charge, which is in agreement with Allers et al.^24^ for NO_2_^+^. Furthermore, the absolute number of O_2_^+^ ions with a maximum total charge of 145 fC at 60 mbar is close to 176 fC at 40 mbar. The relative abundances show a similar behavior compared to the measurements at 40 mbar, despite the missing first ignition region, which may result from the aforementioned subject of averaged ion mobility spectra. When exceeding the maximum total number of ions and transitioning from the glow region to the streamer discharge region, there is a steeper increase of NO_*x*_^+^, a clearly visible decrease in O_2_^+^, but, different compared to 40 mbar, a decrease in H_3_O^+^. Thus, these measurements underline that operation of a corona discharge ionization source in HiKE-IMS should be close to the maximum total charge at the detector for two reasons: (1) absolute numbers of available reactant ions are higher and (2) the most relevant reactant ion species for proton transfer reaction (H_3_O^+^) and for charge transfer (O_2_^+^) are available in large quantities. In summary, all relevant reactant ions in HiKE-IMS are available even at elevated pressures of 60 mbar.

**Figure 9:**
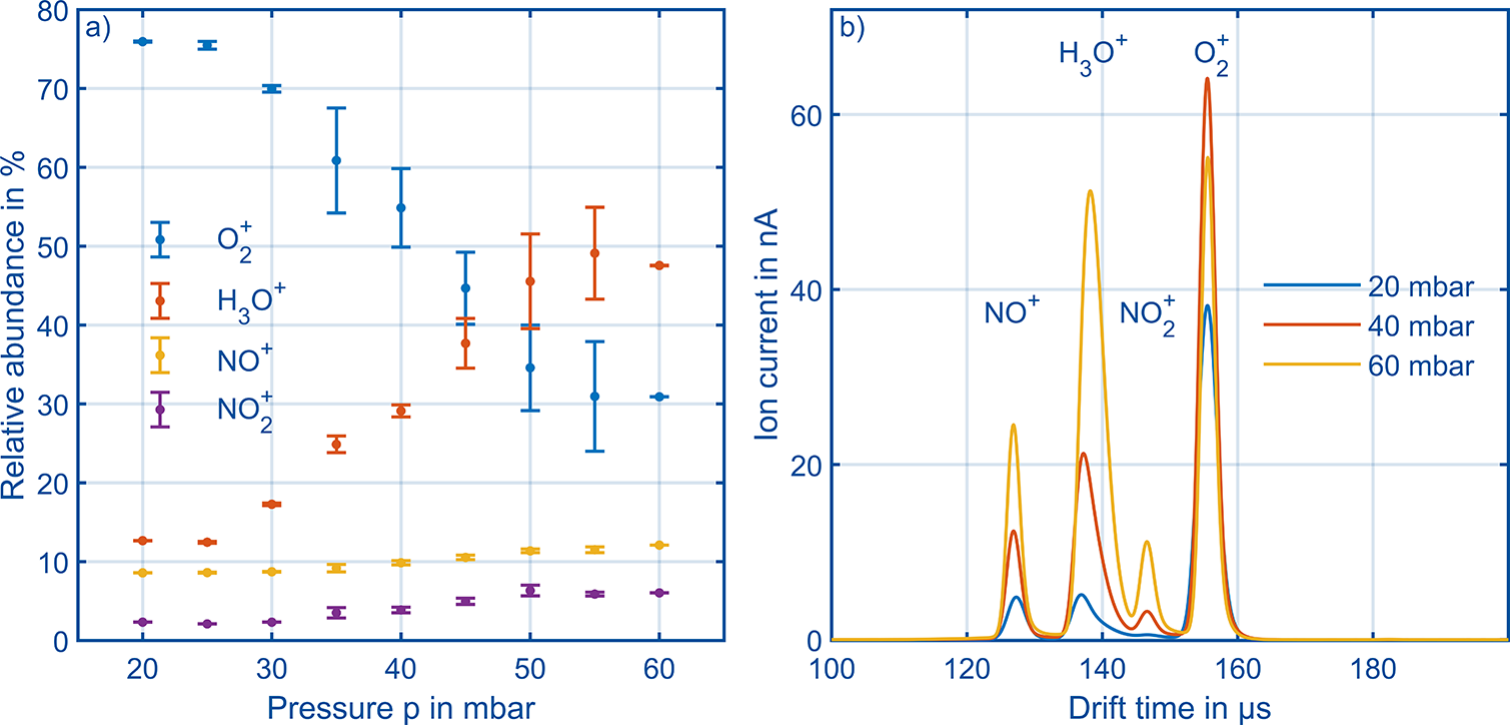
(a) Relative abundance of the four reactant ions in the pronounced maximum at different pressure and same reduced electric field strength ε_SR_ = 100 Td. (b) Reactant ion mobility spectra at three different pressures in the pronounced maximum. Table 1 summarizes all other operational parameters.

Enlarging on the thesis that operation close to the maximum total ion current is favorable, the relative numbers of the different reactant ions at maximum total charge at the detector are plotted over pressure in Figure 9a. Obviously, there is a transition of the most abundant reactant ion, which is from 20 to 45 mbar O_2_^+^ and above 45 mbar H_3_O^+^. Due to increasing pressure and thus neutral gas density, the amount of collisions between reactant ions and neutral water molecules increases, resulting in a conversion of O_2_^+^ to protonated water clusters as shown in refs (22) and (38). This trend is clearly obvious in Figure 9a and can be explained by Reactions 9, 10, and 11 taken from ref (22). There, reactions and reaction rates forming H_3_O^+^ have been collected throughout the literature, the reactions starting with primary ions formed in the corona discharge ionization such as N_2_^+^, which will ionize oxygen via charge transfer as the ionization energy of oxygen (12.07 eV^56^) is lower compared to nitrogen (15.581 eV^56^). This charge transfer reaction is shown in Reaction 9. When O_2_^+^ collides with water, the three body Reaction 10 with the neutral M will result in the formation of O_2_^+^(H_2_O) clusters. When these O_2_^+^(H_2_O) clusters collide with other neutral water molecules, Reaction 11 leads to the formation of H_3_O^+^. Furthermore, we want to note that as shown by ref (22) other reactions are possible also leading toward formation of H_3_O^+^ starting from O_2_^+^ or N_2_^+^.
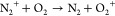
9
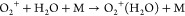
10
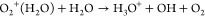
11

Transferring Reaction 11 into a pseudo-first-order differential equation given in eq 12 with the reaction rate constant *k* and the reaction time Δ*t* results in a description of the processes visualized in Figure 9a. Assuming that the concentration of the initially formed ions and thus [O_2_^+^(H_2_O)] is constant at constant ion current, the reaction can be simplified in a first step. As the reaction time Δ*t* at identical reduced electric field strength *E*/*N* stays constant, independently from pressure, and also assuming the reaction rate *k* is also constant, the two variables *k* and Δ*t* can be simplified as the constant *c*. Only the concentration of neutral water molecules [H_2_O] remains as a variable, which is proportional to the pressure *p*. This explains the formation of H_3_O^+^ and decline of O_2_^+^ and its clusters like O_2_^+^(H_2_O), as can be seen in Figure 9a.
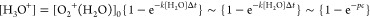
12

Apart from that, the two NO_*x*_^+^ ions will not react with water at reduced electric field strengths above 60 Td, as short reaction times and high kinetic energies prevent cluster formation. Thus, the required cluster NO_*x*_^+^(H_2_O)_*n*_ with cluster size of *n* ≥ 3 is not reached in HiKE-IMS as already shown by ref (22). However, NO_*x*_^+^ also show a slight increase in abundance with pressure up to 6% NO_2_^+^ and around 12% NO^+^. The influence of NO_2_^+^ and NO^+^ is discussed in the next paragraph. Figure 9b shows the reactant ion mobility spectra at maximum charge at the detector for each pressure. The fastest ion is NO^+^, followed by H_3_O^+^, NO_2_^+^, and finally O_2_^+^. Across the different pressures shown here, the O_2_^+^ peak height alternates because of the previously described reactions forming H_3_O^+^, while other peak heights keep growing due to the increasing amount of ions reaching the detector.

**Figure 10:**
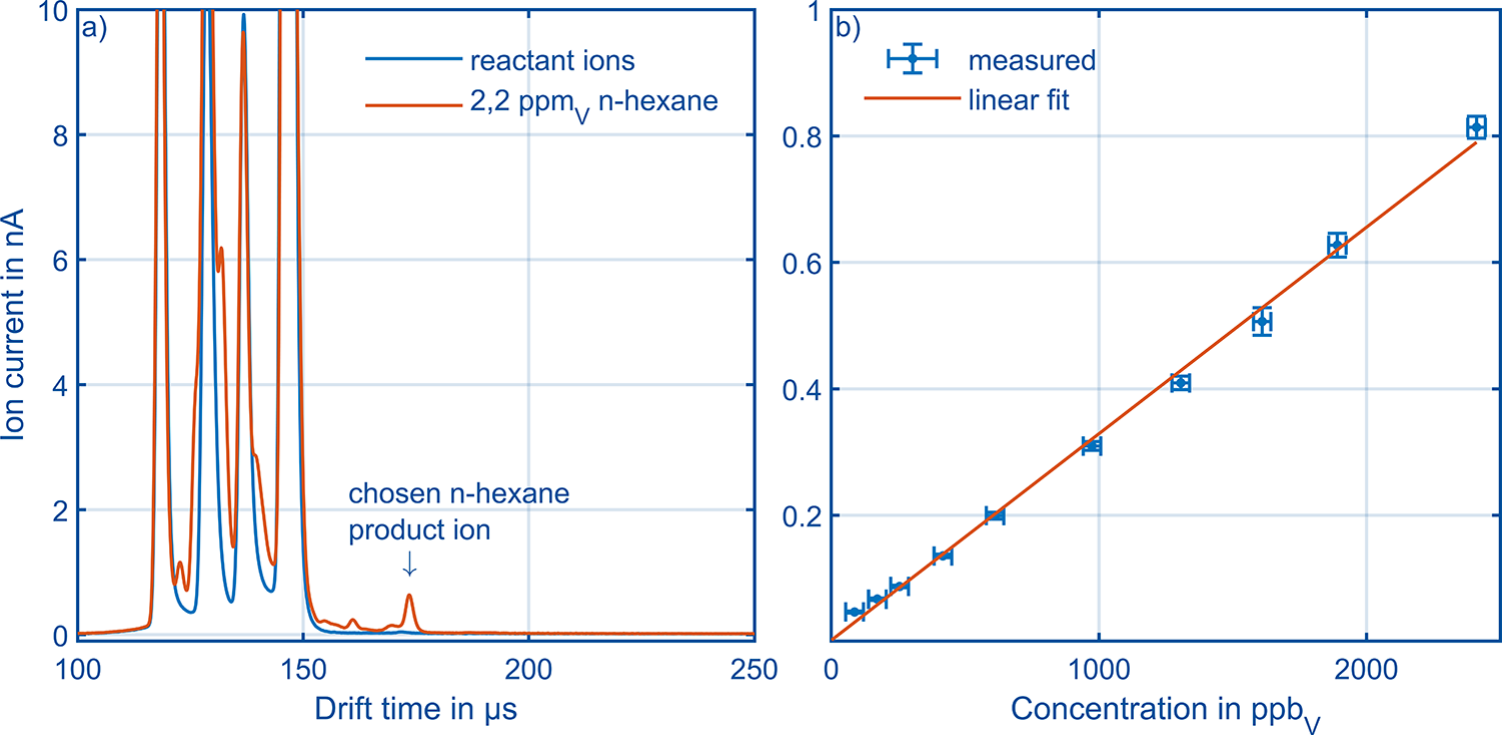
(a) Reactant ion mobility spectrum (blue) of the sample gas and ion mobility spectrum of 2.2 ppm_V_*n*-hexane in the sample gas (red) both at 60 mbar ε_RR_ = 85 Td, ε_SR_ = 105 Td at optimum corona ionization source voltage. Table 1 summarizes all other operational parameters. (b) Calibration curve of *n*-hexane using the settings from Figure 10a and the largest product ion peak at a drift time of 170 μs. Error bars for the concentrations are calculated from the given errors of the flow controllers used for gas dosing (EL-FLOW Select (50, 500, 2000 mL/min), Bronkhorst, Netherlands) and the errors related to calculating the permeation rate from the weight loss of the hexane permeation tube (CPA225D, Sartorius, Germany); error bars for the ion current are calculated by using eight recorded ion mobility spectra per data point. Regarding the concentrations, we assume that adsorption and desorption effects at surfaces can be neglected since all measurements were carried out after reaching constant signals.

In order to demonstrate HiKE-IMS operation at 60 mbar, the carcinogenic *n*-hexane that is difficult to ionize in IMS operated around 1000 mbar is considered as a model substance. Lowest concentrations of *n*-alkanes recorded with corona discharge IMS operated around 1000 mbar are in the region of 1–10 ppm_V_.^57^ For example in the European standard EN 71–9, the maximum emission of *n*-hexane from toys for children is given as 1.8 mg/m^3^ in air (510 ppb_V_), which is below the above-mentioned detection limits for classical IMS. *n*-Hexane can be ionized neither via proton transfer with H_3_O^+^ nor via charge transfer with NO_*x*_^+^ due to their low ionization energies (NO 9.2642 eV, NO_2_ 9.586 eV^56^) compared to 10.13 eV^56^ of *n*-hexane. However, HiKE-IMS allows using O_2_^+^ as a reactant ion even at 60 mbar with an ionization energy of 12.07 eV^56^ for the neutral, sufficiently high for ionizing *n*-hexane. Figure 10a shows the reactant ion mobility spectrum (blue) with the known reactant ions and the ion mobility spectrum of 2.2 ppm_V_*n*-hexane in sample gas (red) resulting in a variety of product ions. The high number of different *n*-hexane product ions can result from three different effects: First, due to the difference in ionization energy between O_2_ and *n*-hexane of 1.94 eV plus additional energy from the reduced electric field strength, fragmentation is possible. Second, high energetic but short-lived ions like N_2_^+^ may reach into parts of the reaction region and directly ionize *n*-hexane due to the high ionization energy of N_2_ (15.58 eV). The energy difference between nitrogen and *n*-hexane of 5.45 eV is higher compared to the oxygen/*n*-hexanes energy difference of 1.94 eV, which should certainly result in fragmentation. Third, if neutral *n*-hexane gets into the corona discharge itself even electrons can ionize *n*-hexane, which would lead to fragmentation of *n*-hexane as known from electron ionization mass spectrometry (EI-MS).^56^ In order to find the special position where *n*-hexane is ionized and by which mechanism, it is necessary to build a new HiKE-IMS with multiple gas inlet and outlet positions. This would be an investigation for a future HiKE-IMS paper. The largest *n*-hexane product ion peak that is well separated from the reactant ions at a drift time of 170 μs was chosen for recording the calibration curve given in Figure 10b. Here, the limit of detection is defined as the product ion peak thrice as large as the noise σ of the averaged ion mobility spectra. HiKE-IMS achieves a limit of detection of 5 ppb_V_ for *n*-hexane within 5 s of averaging.

## Conclusion

In this paper, we discussed how to design a HiKE-IMS with a special focus on its electrodes to achieve high reduced electric field strengths of up to 100 Td at elevated pressures without dielectric breakdown. Hereby, a theoretical approach considering Paschen’s law was chosen to construct new PCB-based HiKE-IMS that can be operated at an absolute pressure of 60 mbar, reaching reduced electric field strengths up to 105 Td. Since HiKE-IMS pressure now covers a pressure range from 7^37^ to 60 mbar, a more detailed investigation into how the used corona discharge ionization source is affected by the operating pressure is possible. The experiments show that there is a maximum total charge reaching the detector. Operation close to this maximum is beneficial as signal intensities are maximized and the relevant reactant ions H_3_O^+^ and O_2_^+^ are available in large quantities. With direct ionization of *n*-hexane from O_2_^+^ a limit of detection as low as 5 ppb_V_ has been reached. Thus, HiKE-IMS operation has now been pushed for the first time to higher pressure of 60 mbar, which allows not only for further miniaturization of the vacuum pump and thus the HiKE-IMS instrument toward a hand-held device, but also for increased sensitivity.
